# Running for Your Life: Metabolic Effects of a 160.9/230 km Non-Stop Ultramarathon Race on Body Composition, Inflammation, Heart Function, and Nutritional Parameters

**DOI:** 10.3390/metabo12111138

**Published:** 2022-11-18

**Authors:** Daniel A. Bizjak, Sebastian V. W. Schulz, Lucas John, Jana Schellenberg, Roman Bizjak, Jens Witzel, Sarah Valder, Tihomir Kostov, Jan Schalla, Jürgen M. Steinacker, Patrick Diel, Marijke Grau

**Affiliations:** 1 Department of Internal Medicine, Division of Sports and Rehabilitation Medicine, University Hospital Ulm, 89075 Ulm, Germany; 2 Department of Central IT, Division Applications and Databases, University of Zurich, 8057 Zurich, Switzerland; 3 Institute of Cardiovascular Research and Sports Medicine, Molecular and Cellular Sports Medicine, German Sport University Cologne, 50933 Cologne, Germany

**Keywords:** ultramarathon, inflammation, body composition, heart function, exercise stress, endurance exercise

## Abstract

Moderate endurance exercise leads to an improvement in cardiovascular performance, stress resilience, and blood function. However, the influence of chronic endurance exercise over several hours or days is still largely unclear. We examined the influence of a non-stop 160.9/230 km ultramarathon on body composition, stress/cardiac response, and nutrition parameters. Blood samples were drawn before (pre) and after the race (post) and analyzed for ghrelin, insulin, irisin, glucagon, cortisol, kynurenine, neopterin, and total antioxidant capacity. Additional measurements included heart function by echocardiography, nutrition questionnaires, and body impedance analyses. Of the 28 included ultra-runners (7f/21m), 16 participants dropped out during the race. The remaining 12 finishers (2f/10m) showed depletion of antioxidative capacities and increased inflammation/stress (neopterin/cortisol), while energy metabolism (insulin/glucagon/ghrelin) remained unchanged despite a high negative energy balance. Free fat mass, protein, and mineral content decreased and echocardiography revealed a lower stroke volume, left end diastolic volume, and ejection fraction post race. Optimizing nutrition (high-density protein-rich diet) during the race may attenuate the observed catabolic and inflammatory effects induced by ultramarathon running. As a rapidly growing discipline, new strategies for health prevention and extensive monitoring are needed to optimize the athletes’ performance.

## 1. Introduction

Although humanity has evolutionarily developed to be a race of two-leg runners that can sustain running potentially for days [1], the impact of long distance runs on the human body is still largely unknown. An ultramarathon is a modern type of long-lasting competition and includes all distances above 42.195 km, the traditional marathon distance [2]. In recent years, this discipline has enjoyed growing popularity and has come into focus of exercise research [3]. Although regular endurance exercise at moderate intensity is accepted as “natural medicine” for longevity and health risks’ reduction [4,5], it remains unknown whether an ultramarathon—which usually takes at least 6 h from start to finish—and its high training volumes may present health risks [6,7].

Strenuous long-term endurance exercise in training and/or competition induces changes in metabolic demands and may cause long-lasting disturbances in the individual hormonal profile [8]. These disturbances can contribute to an increased risk of cardiovascular, respiratory, musculoskeletal, renal, immunological, gastrointestinal, or neurological injuries [8,9]. Even the heart seems to be transiently affected, with up-to-now no definite conclusions on the immediate health outcomes [10,11]. Further risk factors, depending on the environmental conditions, include fluid/electrolyte disturbances, central nervous system, and gastrointestinal system problems, as well as dehydration and exercise-associated muscle disturbances [12].

Besides the obvious orthopaedical demands of an ultramarathon, including musculoskeletal injuries [13], the stress-induced damages on the molecular and cellular level are equally challenging to the athletes’ health and regeneration. Inflammatory processes can be observed after a competition with an increase in pro-inflammatory cytokines [14] and oxidative stress [15], which can last for several days or months, along with reduced anti-oxidative capacity [16]. Even the gut microbiota seems to be negatively affected after an ultramarathon competition, which may compromise the athletes’ immune function [17]. The assessment of the stress response on the molecular level is thus of the utmost importance to reduce the health risk during participation in ultramarathons, but is still underrepresented in current studies. Monitoring the depletion of anti-oxidative capacities, the changes in stress markers like cortisol or the measurement of immunological risk markers like neopterin or kynurenine may improve the present gaps in molecular adaptations.

A further consideration for ultramarathon performance capacity is the balance of energy expenditure and consumption. Studies revealed a runner’s energy deficit of about 7000 kcal (or 30 MJ) per day, despite a high amount of food and (energized) water intake [18,19]. This deficit might subsequently put health at risk and increase the risk of injury; especially in multistage races, e.g., through a continuous catabolic state [6,9,20]. Nutritional strategies during the race might vary and depend on personal experience rather than official recommendations, partly owing to limited scientific evidence or insufficient transfer from the scientific into the broad running community [9]. Knowing that gastrointestinal symptoms are one of the most common problems experienced during ultramarathon races [6,18], optimization of nutritional strategies for energy refueling with concomitant maintaining good gastric tolerance and high nutrient density intake is a challenging task. Furthermore, hunger signaling is reduced during and shortly after endurance exercise, which can intensify reduced energy intake [21,22], and studies examining energy related hormonal responses or e.g., gluconeogenesis, during ultra-endurance events are missing.

Despite that, studies suggest that especially well-trained competitors may compensate for the long-lasting physiological load and stress through an improved cardiovascular system [23,24], cognitive abilities [25], and lipid profile [26]. These seemingly contradicting observations of the health risks and potential benefits of ultramarathon training and competition require investigations regarding the impact of long-lasting exercise characterized by relatively low intensities.

The TorTour de Ruhr^®^, which is the focus of the present investigation, is a non-stop ultramarathon that takes place every two years in Germany and includes the distances 100 km, 160.9 km, and 230 km. The start–finish of the 230/160.9 km race is Winterberg–Duisburg and Arnsberg–Duisburg, respectively. It is one of the most physiologically and psychologically demanding runs in Germany that requires optimal performance capacities. This study aimed to assess body composition, antioxidant status and cardiac/metabolic stress, and energy parameters before (pre) and after the race (post) to gain new insights into the effects of this extreme endurance race regarding molecular mechanisms and interactions that might help to improve performance capacity and to protect the health of the participants.

## 2. Materials and Methods

### 2.1. Entry Eligibility

TorTour de Ruhr^®^ participation required a medical sports examination that had been conducted less than 6 months before the race and that confirmed the physical resilience of the athlete.

Inclusion criteria for the study participation were as follows: male/female; endurance athlete and 160.9 km or 230 km participant of the TorTour de Ruhr^®^ 2022; no previous injuries; and the ability to understand the study procedure and to give informed consent.

Exclusion criteria included the following: participants of the 100 km (owing to organizational restrictions); nicotine consumption; diseases of the intestine; blood clotting disorders or intake of blood-thinning medications; acute or chronic vascular (blood flow) disorders; cardiovascular, metabolic, or autoimmune diseases; and non-consenting subjects.

All subjects received information about the study content and the use of the data and provided written consent. The study was conducted in compliance with the Declaration of Helsinki. The study was approved by the ethics committee of German Sports University Cologne (25/2020).

All measures included pre and post assessments. Pre-race measurements were carried out the evening (230 km) or two hours (160.9) before the start at the organizer’s race briefing to determine basal resting values, while post-race measurements were performed immediately at the finish line at the finishers’ arrival (Supplemental File S1, Figure S1). The study team was divided into groups taking (i) body composition, (ii) echocardiographic and (iii) laboratory data, and (iv) blood sampling to minimize examination time pre and post. Blood sampling as well as echocardiography was performed by the study physician with an MD degree in internal medicine and sports medicine as well as a special expertise in cardiology. Blood samples were taken pre and post from the *vena mediana cubiti* and anticoagulated using ethylenediaminetetraacetic acid (EDTA) as anticoagulant. Blood samples were stored at 4 °C until transportation to the analysis facility. All samples were transported in a time frame of a maximum of four hours and either measured immediately in the laboratory or stored at −80 °C until further analysis.

### 2.2. Anthropometry and Body Composition

Anthropometric measurements included height, body mass, and body composition. Height was measured without shoes, in light clothing, with a standardized scale. For measuring body mass and body composition, a bio-impedance scale (InBody 770, InBody Europe B.V., Eschborn, Germany) was used.

### 2.3. Study Group Characteristics

In total, 28 ultramarathon runners (7f/21m) were initially included. Participants had already completed 62 ultramarathons on average. Twelve participants dropped out during the race. From the remaining 16 finishers (4f/12m), 4 did not finish within the defined period (cut-off time: 38 h/230 km and 30 h/160.9 km). Data of a total of 12 (2f/10m) participants were finally analyzed. Detailed anthropometric characteristics of the runners are presented in Table 1.

### 2.4. Environmental Conditions

Blood sampling of the 230 km race was scheduled for 3 June 2022, between 6 and 8 p.m., while blood sampling of the 160.9 km race was scheduled for 4 June 2022, between 4 and 5:30 p.m. The start of the 230 km race was scheduled for 4 June 2022, at 8 a.m., at 13.8 °C with a humidity of 71.6% and 14.4 km/h west-wind. The start of the 160.9 km race was scheduled for 4 June 2022, at 6 p.m.; the temperature was 23.1 °C, humidity 50%, and wind 7.2 km/h south-west. The finish was 5 June between 10:30 a.m. and 8:00 p.m.; the mean temperature during participants’ arrival was 21 °C, with a humidity of 83%, occasional rain, and 18 km/h south-west wind.

### 2.5. Energy Intake and Expenditure

Participants received a questionnaire and were asked to note the time of food intake and a detailed description of the respective meal/component. Briefly, the amount of food/snack (in grams/household measurements, i.e., one handful, one bowl, cup, mug, and so on), the fat content for dairy products, the respective brand, all beverages (quantity and brand of beverage, if applicable), and the mixing ratio for homemade drinks should be indicated. Documentation was performed by the mandatory athletes’ team crew or retrospectively by the athlete himself/herself. In addition, the questionnaire included questions regarding gender, age, number of ultramarathons, type/amount/number of supplements, specialized nutritional habits (e.g., vegan/vegetarian), experienced gastrointestinal symptoms related to an ultramarathon, and products that are repeatedly/always used for food during ultramarathons.

For evaluation of the total energy intake and the respective contribution of protein/fat/carbohydrates, EBIS Pro software (EBIS Pro 3.02, http://ebispro.de/, accessed on 1 November 2022) was used.

The energy expenditure calculation was based on the heart frequency determined by the respective running wearable (Garmin International, Olathe, KS, USA) during the race and the respective energy expenditure data on the Garmin Connect online platform.

### 2.6. Energy Metabolism and Stress Response

Ghrelin (#BMS2192, Thermo Fisher, MA, USA), insulin (ab100578, Abcam, Berlin, Germany), irisin (EK-067-29, Phoenix Pharmaceuticals, Burlingame, CA, USA), glucagon (#EHGCG, Thermo Fisher, MA, USA), cortisol (#15642299, Fisher Scientific, Reinach, Switzerland), neopterin (#RE59321, IBL International, Hamburg, Germany), and total antioxidative capacity (TAC, measurement of the combination of both small molecule antioxidants and proteins by Cu^2+^ reduction) (ab65329 Abcam, Berlin, Germany) were analyzed in the plasma fraction using respective enzyme-linked immunosorbent assays (ELISA) according to the manufactures’ instruction.

Plasma kynurenine concentrations were determined by spectrometry using an established protocol [27]. Briefly, serum samples were deproteinized with acetic acid trichloride and following deproteinization. Kynurenine reacts under the use of 4-dimethylamino-benzaldehyde into a yellow product, which can be measured spectrometrically at 492 nm and quantified with concomitant preparation of a stand with known concentrations.

Tissue glucose was measured with an intracutaneous glucose sensor (Free style libre 3, analyzed by App-based program LibreView, Abbot Diabetes Care, Alameda, CA, USA).

### 2.7. Echocardiography

Echocardiographic examinations were performed using an CX50 ultrasound system with a phased-array probe S5-1 (Philips GmbH, Hamburg, Germany). The images were analyzed offline using TomTec post-processing software (2D Cardiac Performance Analysis, TomTec Imaging Systems, Unterschleissheim, Germany). Measurements were made in the apical four-, three-, and two-chamber views and in the parasternal short and long axes. The following parameters were collected: left ventricular ejection fraction (EF by biplane LV planimetry by the modified Simpson’s rule and EF two-dimensional by Teichholz), end-diastolic volume (EDV), end-systolic volume (ESV), and stroke volume (SV). The wall thicknesses of the interventricular septum and left ventricular free wall in diastole (IVSd/LPWd) and systole (IVSs/LPWs) as well as the left ventricular diameter in diastole (LVIDd) and systole (LVIDs) and left atrial size (in mm) and diameter of the aortic root were determined. Diastolic function was characterized by maximum velocities of E and A waves (V_max_E, V_max_A), E/A ratio, E/E’lateral ratio (V_max_E and maximum myocardial velocities (E’lateral) of the lateral mitral annulus), E/E’medial ratio (V_max_E and maximum myocardial velocities (E’medial) of the basal mitral annulus), and deceleration time (dec time). Right ventricular diameter diastolic (RVIDd), right ventricular function (tricuspid annular plane systolic excursion (TAPSE), and maximum velocity across the tricuspid valve (TVmax) and maximum pressure gradient (TVmax PG) were determined. The endocardial contour was manually adjusted. Images that did not capture all myocardial segments sufficiently well and participants from whom only one examination (pre or post) was available were excluded from the analysis. To avoid interrater variability, the echocardiographic analysis was performed by one investigator.

### 2.8. Statistics

Data analysis was performed using GraphPad Prism (GraphPad Prism 9.4; San Diego, CA, USA). To improve data analysis and statistical power, data of both 160.9 and 230 km participants were analyzed in one dataset. Subgroup analysis was performed for descriptive statistics (anthropometry, energy expenditure/intake, and finish time). All data were tested on Gaussian distribution using the Kolmogorov–Smirnov normality test. One-tailed t-testing was used comparing pre and post for all normally distributed data. Otherwise, a Wilcoxon matched-pairs signed rank test was used to determine the statistical significance of differences between pre and post. If not other stated, all data are presented as mean ± standard deviation. Statistical significance was established at *p* ≤ 0.05.

## 3. Results

### 3.1. Race Nutrition

In this study, 36% of the participants reported the regular intake of dietary supplements (mainly micronutrients/vitamins, sports refresher, or protein powder), 46% did consume special supplements, and 18% provided no information. Mean heart frequency during the race was 120 ± 10 beats per minute. Mean energy deficit was calculated to be around 6000 kcal (160.9 km) and 9000 kcal (230 km), respectively. The detailed energy expenditure and food intake as well as the macronutrient distribution are presented in Table 1. Energy-providing components of the diet during the race consisted of 8.8 ± 4.1% protein, 24.4 ± 16.4% fat, 64.6 ± 17.5% carbohydrates, and 2.2 ± 2.4% alcohol.

### 3.2. Body Composition

**Figure 1:**
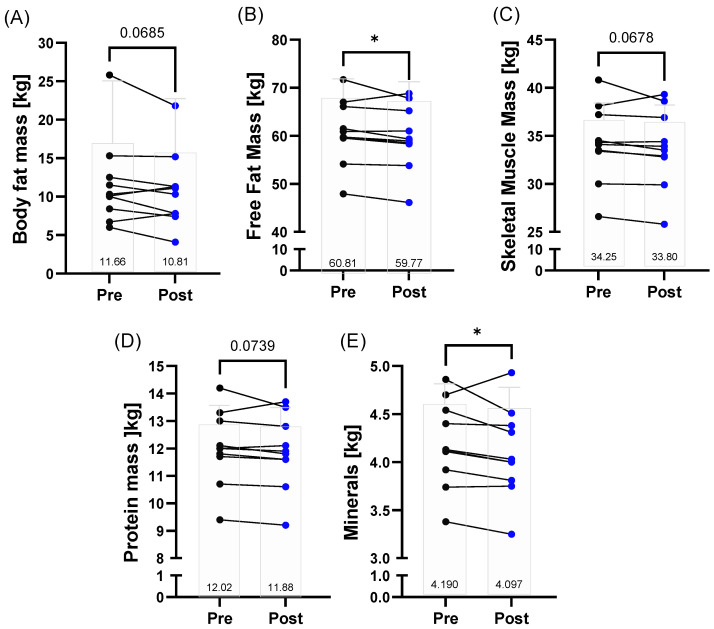
Body composition variables related to tissue distribution and micronutrients of the 160.9/230 km finishers before (pre) and after (post) the race (full dataset obtained for *n* = 9). The decrease in (**A**) body fat mass, (**C**) skeletal muscle mass, and (**D**) protein mass was on the verge of statistical significance. (**B**) Free fat mass and (**E**) mineral content were significantly decreased post-race. Individual changes as well as mean number values are presented. * *p* ≤ 0.05.

Body fat mass (*p* = 0.0685), free fat mass (*p* = 0.0277), and skeletal muscle mass (*p* = 0.0678) were reduced post-race. While there was only a trend for decreased total protein mass post-race (*p* = 0.0739), mineral content was significantly lower after the race (*p* = 0.0425) (Figure 1A–E).

### 3.3. Energy Metabolism

**Figure 2:**
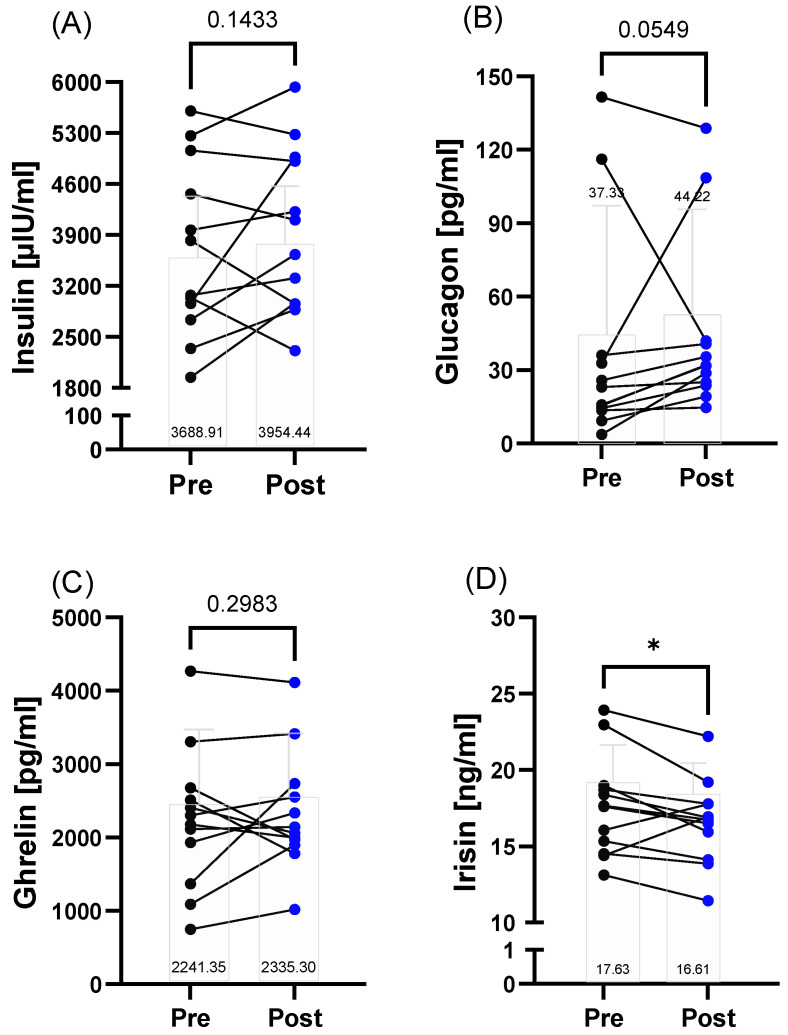
Energy- and nutrition-related variables in the 160.9/230 km race finishers (*n* = 12) before (pre) and after (post) the 160.9/230 km competition. (**A**) Individual insulin concentrations showed high variation and mean values did not significantly change from pre to post (**B**). Glucagon concentration was higher post-race. (**C**) Ghrelin values remained unchanged from pre to post, while (**D**) irisin levels decreased post-race. Presented are individual changes as well as mean number values. * *p* ≤ 0.05.

Energy-related and nutrition-dependent variables insulin (*p* = 0.1433), glucagon (*p* = 0.0549), and ghrelin (*p* = 0.2983) were not significantly different from pre to post, but showed highly individual variability. Irisin decreased post-race (*p* = 0.0324) (Figure 2A–D).

Despite infrequent and various energy intake, tissue glucose concentrations determined in a subgroup of participants (*n* = 4) never reached values below (70 mg/dL) or above (180 mg/dL) reference values (Supplementary File S1, Figure S2).

### 3.4. Stress Response

**Figure 3:**
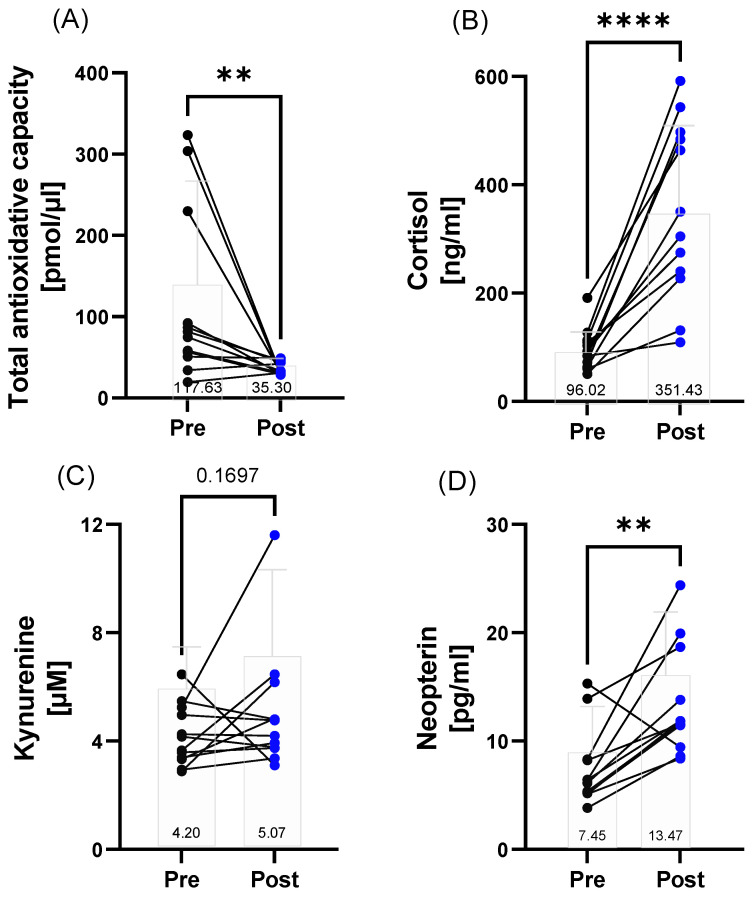
Stress response and immunological variables in the 160.9/230 km race finishers (*n* = 12) before (pre) and after (post) the 160.9/230 km competition. (**A**) Total antioxidative capacity decreased post-race and (**B**) cortisol levels increased after the race. (**C**) Kynurenine concentrations remained unchanged. (**D**) Neopterin levels increased post-race. Presented are individual changes as well as mean number values. ** *p* ≤ 0.01; **** *p* ≤ 0.0001.

Total antioxidative capacity decreased post-race (*p* = 0.0024), while the cortisol concentration (<0.0001) and neopterin (*p* = 0.0034) level increased. Kynurenine concentrations (*p* = 0.1697) remained unchanged during the race (Figure 3A–D).

### 3.5. Echocardiography

**Figure 4:**
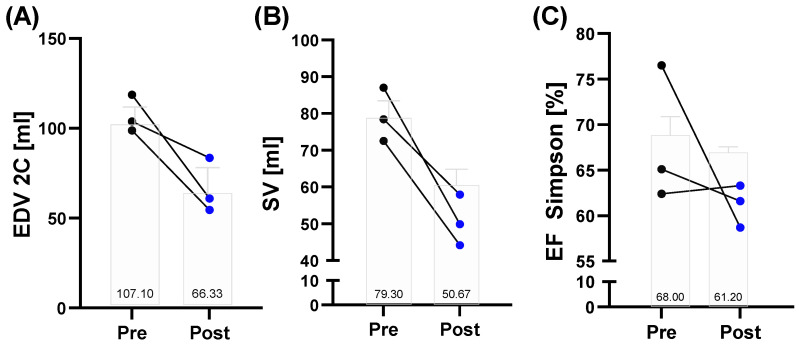
Selected echocardiographic data of a sub-group of the 160.9/230 km race finishers (*n* = 3) before (pre) and after (post) the 160.9/230 km competition. (**A**) Left-end diastolic volume (EDV) and (**B**) the stroke volume (SV) decreased post-race in all analyzed finishers. (**C**) Ejection fraction was markedly decreased in one participant. Presented are individual changes as well as mean number values.

In a sub-group of *n* = 3, a -re–post comparison of echocardiographic data was obtained (Supplementary File S2, Figures S1 and S2). In all three individuals, a decrease in end-diastolic volume (EDV; mean reduction from 107.1 mL to 66.3 mL, 38%) and stroke volume (SV; mean reduction from 79.3 mL to 50.7 mL, 36%) was observed, while one participant showed a reduction in the ejection fraction from pre (76.5%) to post (58.7%) (Figure 4A–C).

## 4. Discussion

While ultramarathon running has gained increasing interest, with increasing numbers of participants, the effects of such a long but psychological/orthopedic rather than cardiovascular demanding exercise are scarcely examined. Optimizing performance and nutrition strategies in races over 50 km is paramount for reducing health risks and improving race outcome and recovery. We found that a non-stop ultramarathon of 160.9 km or 230 km induces a severe mismatch of energy expenditure and consumption that may contribute to a catabolic state on the muscular and protein level. However, the diet as well as the hormonal response of the participants related to energy metabolism seem to be highly individual, whereas a clear depletion of antioxidative capacities and concomitant stress response was observed.

With increasing age, a decline in running speed and contractile performance is inevitable [28]. Nevertheless, continuous training maintains a high aerobic potential with the capacity to run for a long distance at an even high pace [28]. Besides the mental toughness to finish in such competitions, the physical state and experience are the prerequisites for a successful finish. The mean age of the recent participants was about 50 years and they reported a decade-long experience in long distance running. Although the maximal heart rate is highly individual and declines with age [29,30], the observed mean heart rate of 120 beats per minute during the race suggested a moderate running intensity and thus, on the first sight, a more musculoskeletal rather than metabolically challenging competition.

### 4.1. Energy Metabolism and Nutritional Strategies

Long distance runs are challenging in terms of energy balance. The individual energy intake calculated in the present study was insufficient to balance the energy consumption, and the nutritional composition was inadequate because a loss of protein, skeletal muscle, and minerals was determined. The mean energy deficit was calculated in the range of 6000 kcal (160.9 km) to 9000 kcal (230 km), with the results thus comparable to other ultramarathon studies [18,19]. According to the analyzed nutrition protocols, the nutrition during the race was high in carbohydrates and fat, whereas the relative protein concentration was rather low. Although carbohydrates and fat are the main energy contributors at moderate intensities [31], the low protein intake of around 8.8% might be one contributor of the catabolic muscle state observed after finishing the race. A recent review summarized the different energy contributors during an ultra-endurance event and indicated that the percentage of the respective fat, carbohydrate, and protein consumption highly varies depending on race distance (100–1000 km, mostly carbohydrates) and environmental conditions (e.g., Antarctic race, mostly fat) [18]. Current consensus guidelines suggest a macronutrient distribution of 60% carbohydrates, 15% protein, and 25% fat during ultramarathon training, while a high contribution of carbohydrate and fat intake should be preferred during racing, with no special percentage recommended [9]. Thus, the present athletes met the criteria for training sessions during the race, which further underlines the individual nutritional strategies relying on personal experience.

The hormonal, energy-related response regarding insulin (glucose uptake), glucagon (glycogenolysis), and ghrelin (“hunger signaling”) showed a highly individual pattern, including either slightly decreasing, increasing, or even unchanged values. It was only possible to obtain full datasets of *n* = 4 participants regarding glucose sensor measurements during the race. However, the monitored glucose levels remained within reference ranges during the competition and are thus in accordance with the measured insulin and glucagon levels, indicating a rapid and constant glucose uptake and energy refueling after energy uptake. These are the first data reporting the influence of a 160.9/230 km race on energy and metabolic markers, thus comparison to the recent literature is hardly possible. Yet, a study by Arakawa et al. examined insulin, leptin, and adiponectin during a 2-day long 130 km ultramarathon and the results also indicated individual patterns at the different examination time points [32]. Another study examined energy and bone metabolism in 17 trained runners after a 65 km mountain ultramarathon and suggested unchanged insulin and ghrelin levels pre to post race, but increased glucagon values as well as lower leptin levels [8]. The authors concluded that maintaining energy homeostasis during the race induced detrimental effects on bone metabolism owing to the high amount of energy needed. The metabolic disturbances could be one cause for the decrease in irisin levels observed in our athletes after the race. The main function of the myokine irisin is thermogenesis regulation, inducing hormone release for increasing energy expenditure, promotion of weight loss, and decreased insulin resistance [33]. To the best of our knowledge, irisin levels were only measured during the possible coldest ultramarathon, the Yukon Arctic Ultra, with slightly increased values measured after the race [34]. If this external stimulus of cold exposure is missing, it may be assumed that the stressful exertion in a warm environment like in our present study induced the opposite effect of irisin depletion rather than irisin expression without performance benefits.

### 4.2. Inflammatory and Immunological (Stress) Response

Ultramarathon running evokes an increase in pro-inflammatory cytokines like interleukine-6, interleukine-8, tumour-necrosis-factor-α, and c-reactive protein [35]. Hohl et al. observed an increase in the concentration of the stress marker cortisol and c-reactive protein levels after a 24 h ultramarathon [36]. The data of the present investigation confirmed the increase in cortisol levels after the finish and further revealed a significant depletion of total antioxidative capacity in the investigated athletes as well as higher neopterin concentrations. Neopterin, which belongs to the chemical group known as pteridines, is synthesized by human macrophages upon interferon-gamma stimulation. It is indicative of a pro-inflammatory immune status and thus serves as a marker of cellular immune system activation [37]. Increased neopterin values were reported by Lucas et al. during a 90 km long Brazilian ultra-endurance event alongside with systemic inflammation and oxidative stress after the race [15,16]. The results of the present study confirmed an acute decrease in the total antioxidative capacity after the race. This finding in combination with the observed pro-inflammatory and immunological response might result in a higher susceptibility for possible infections. On the other hand, kynurenine (a downstream key player of the tryptophan metabolism and upregulated, e.g., in mental disorders, immunological stress responses, and overtraining syndrome) [27,38] remained unchanged, suggesting adapted training status of the participants by their decade-long running experience.

Likewise, the increased cortisol levels may be a sign of the adaptation to the physiological load imposed by the continuous exercise. It was observed that the metabolic exercise stress potently stimulated the hypothalamo–pituitary adrenal axis, which results in the release of cortisol, which in turn increases the bioavailability of metabolic substrates for muscular energy supply of muscles, normal vascular integrity, and protection from an overreacting immune system [26,39,40]. In addition, it is known that heavy continuous exertion results in a strong pro-inflammatory response, whereas concomitant increases in anti-inflammatory hormones like cortisol or interleukin-1 receptor antagonist suggest a counter-reacting immune system in athletes [41]. This response can be modulated by training status and exercise type and experience, e.g., long-distance runners showing an attenuated cortisol release compared with middle-distance runners [42].

While high individual variation or no response were observed for energy-related metabolites, parameters reflecting the immune and stress system were comparable for all runners. This in part confirms the results of Jastrzębski et al., who observed that negative metabolic responses are independent of runners´ age and running experience [43]. It has to be mentioned that the study by Jastrzębski et al. covered a running distance of 100 km, with a mean finish time that was only half (160.9 km) or one-third (230 km) of our finishers. Thus, the stress responses are not fully comparable and require further investigation as well as the individual adaptation of male and female competitors, who seem to have different stress responses on the muscular, cardiovascular, and cardiac level [44].

### 4.3. Cardiac Considerations

The cardiac pre to post stress response could only be obtained from three participants owing to technical and organizational restraints at the finish. Nevertheless, individual analysis showed decreased left end diastolic volume, a reduced stroke volume, as well as lower ejection fraction in one individual. These findings in part confirm the results of Lord et al., who examined right and left ventricular function in ultramarathon runners (100 mile race). The authors described a rightward shift in the right ventricular (RV) area-ε loop as a result of RV dilatation with a concomitant leftward shift in the left ventricular area-ε loop as a result of underfilling of the left ventricle. The parameters of the left ventricle were in part restored six hours after the finish, while RV function remained depressed. The resulting long-term consequences of these diametric developments remain inconclusive [11], but the reduced stroke and end diastolic volume in our runners showed at least acute impaired heart function immediately after the race. In addition, a study on the effects of a 246 km ultramarathon on cardiac strain described that the ventricular global longitudinal strain (GLS) as well as effective diastolic period slightly decreased, whereas left atrial strain and inter-chamber relationships remained unchanged. The authors suggested that these changes may be attributed to concomitant pre- and afterload alterations following an ultramarathon [10], which may exert in the end—together with metabolic adaptations in well-trained competitors—even cardio-protective effects [23]. In addition, pre to post examinations of master athletes running an ultramarathon (50 km) did not exhibit any acute exercise-induced atrial dysfunction or exercise-induced supraventricular arrhythmias [45]. Thus, an acute atrial dysfunction seems an uncommon event, at least at shorter ultramarathon distances. Monitoring the heart response in follow-up examinations over several days and measurements of established cardiovascular related metabolites (e.g., Troponin-T, creatin) may add further insights into the possible either detrimental or protective effect of heart function adaptations seen after ultramarathon running.

### 4.4. Limitations

Both the energy intake and expenditure represent estimations based on race protocols (intake) and wearables/algorithms basing on heart rate (expenditure). However, notes of the study participants were as diligent as possible and the calculated negative energy balance reflects data reported by comparable studies. Thus, good validity of the results might be assumed. Measurement of body compositions using the BIA method might be influenced by water intake and previous exercise. To limit the influence of any external factors, the measurements were performed using the same device under comparable conditions. In addition, we observed a drop-out rate of over 50% in this study, which is not unusual at such an event. Thus, only full datasets of *n* = 12 participants were obtained, showing high inter-individual variation for most of the variables, excluding glucose and echocardiographic measurements. Determination of the left ventricular global longitudinal strain was unsuccessful because of too poor image quality in limited pre- and post-race examination conditions. Nevertheless, comparable studies report similar problems.

## 5. Conclusions

Ultramarathon competitions are becoming increasingly popular considering the increasing number of male and female participants. Because the impact of these ultramarathons on orthopedic, cardiovascular, and metabolic structures is largely unknown, research is necessary to investigate possible consequences. The preparation of the athletes for such a competition is highly individual and determined by personal experience rather than scientific evidence. The results of the present study suggest that optimizing nutrition (preparing and planning a high-density protein-rich diet) during a competition may attenuate the observed catabolic and inflammatory effects induced by ultramarathon running, which may result in decreased acute and long-term health risk with possible positive implications on metabolic, muscular, and cellular regeneration. Regarding the rapidly developing technological possibilities (e.g., running wearables, super shoes, and continuous micronutrient monitoring), further studies should examine the usefulness of these available and prospective technologies in combination with the individual athlete’s experience for performance enhancement and risk reduction.

## Figures and Tables

**Table 1:** Anthropometric data, finish time, and nutrition details of the study participants. All data are presented as mean standard ± deviation.

Anthropometry							
Age [years]		Height [cm]		Body Mass [kg]		Body Mass Index [kg/m²]	
160.9 km	230 km	160.9 km	230 km	160.9 km	230 km	160.9 km	230 km
50.20 (7.98)	50.14 (9.74)	174.80 (4.62)	177.43 (8.74)	71.14 (4.18)	71.89 (11.30)	23.30 (1.29)	22.79 (2.93)
**Finish time [hours]**							
**160.9 km**				22.56 (3.31)			
**230 km**				32.28 (2.95)			
**Race Nutrition**							
**Energy expenditure** **[kcal]**		**Energy intake** **[kcal]**		**Macronutrient distribution** **(%)**			
**160.9 km**	**230 km**	**160.9 km**	**230 km**	**Carbohydrates**	**Fat**	**Protein**	**Alcohol**
11,203.78 (1793.33)	15,977.52 (2351.22)	5379.10 (1460.69)	6695.85 (3748.86)	64.6 (17.5)	24.4 (16.4)	8.8 (4.1)	2.2 (2.4)

## Data Availability

All data that are not provided in the main manuscript or Supplementary Files can be provided by the corresponding author upon reasonable request. Not all data are not publicly available owing to privacy restrictions.

## Supplementary Materials

The following supporting information can be downloaded at https://www.mdpi.com/article/10.3390/metabo12111138/s1. Supplemental File S1 Figure S1: Overview of the different data collection time points and respective measurements; Supplemental File S1 Figure S2: Tissue glucose measurement with the Free Style Libre 3 during the TorTour de Ruhr 2022; Supplemental File S2 Figures S1 and S2: Echocardiographic data from a sub-sample of three participants before (pre) and after (post) finishing the 160.9 km/230 km ultramarathon, TorTour de Ruhr 2022. Jana Schellenberg, Jan Schalla.
