# Supplementary material for: Running for Your Life: Metabolic Effects of a 160.9/230 km Non-Stop Ultramarathon Race on Body Composition, Inflammation, Heart Function, and Nutritional Parameters
Source: Metabolites. 2022 Nov 18;12(11):1138. doi: 10.3390/metabo12111138 (PMC9692917; doi:10.3390/metabo12111138)
Supplement: Supplementary file 1 [file metabolites-12-01138-s001.zip › Supplemental S1_rev.pdf]

Supplemental S1: Overview of the different data collection time points and respective measurements

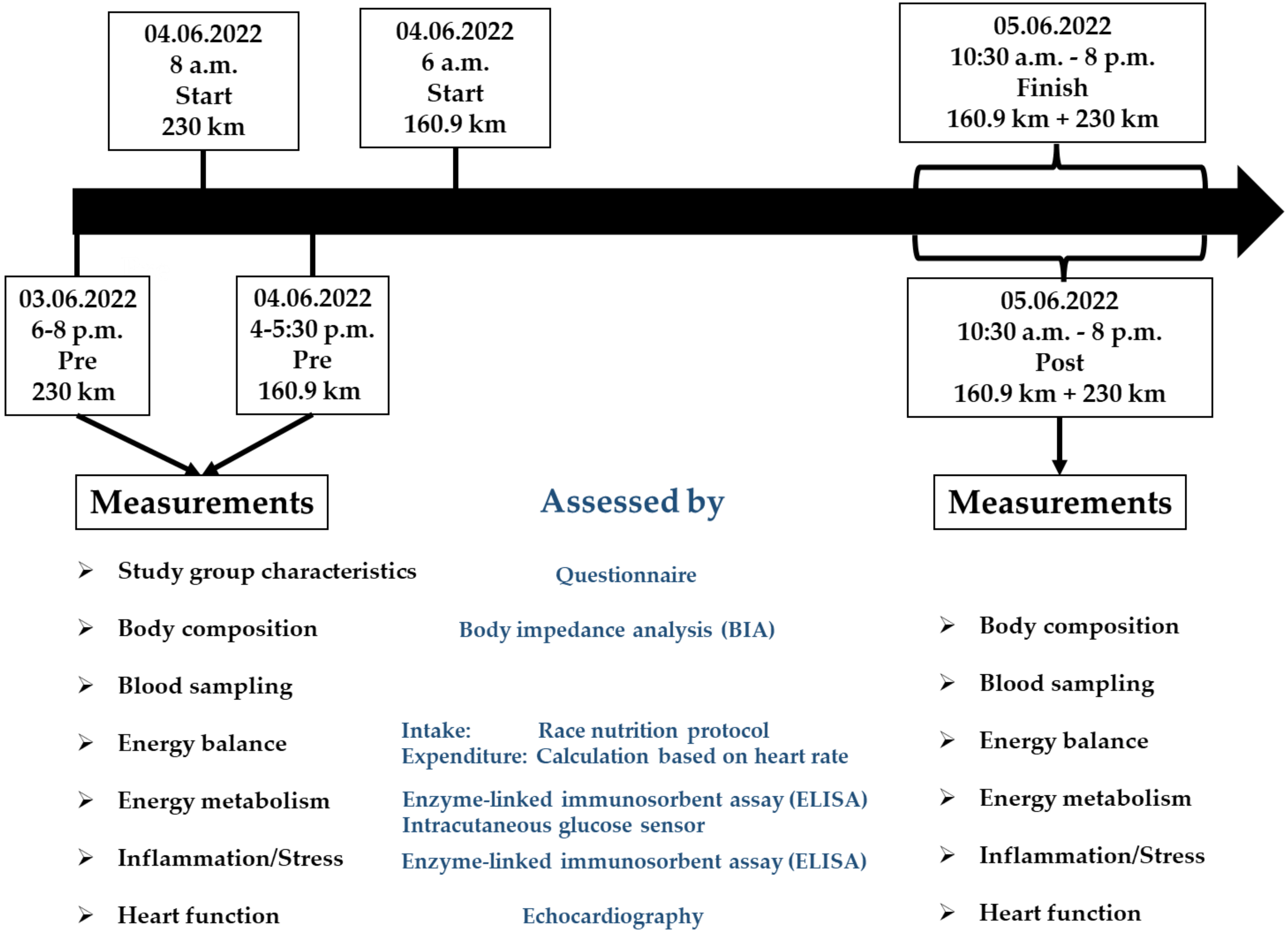

Figure S1: Overview of race start/finish and data collection time points as well as measurement methods for the respective parameter.

Supplemental S1: Tissue glucose measurement with the Free Style Libre 3 during the TTdR2022.

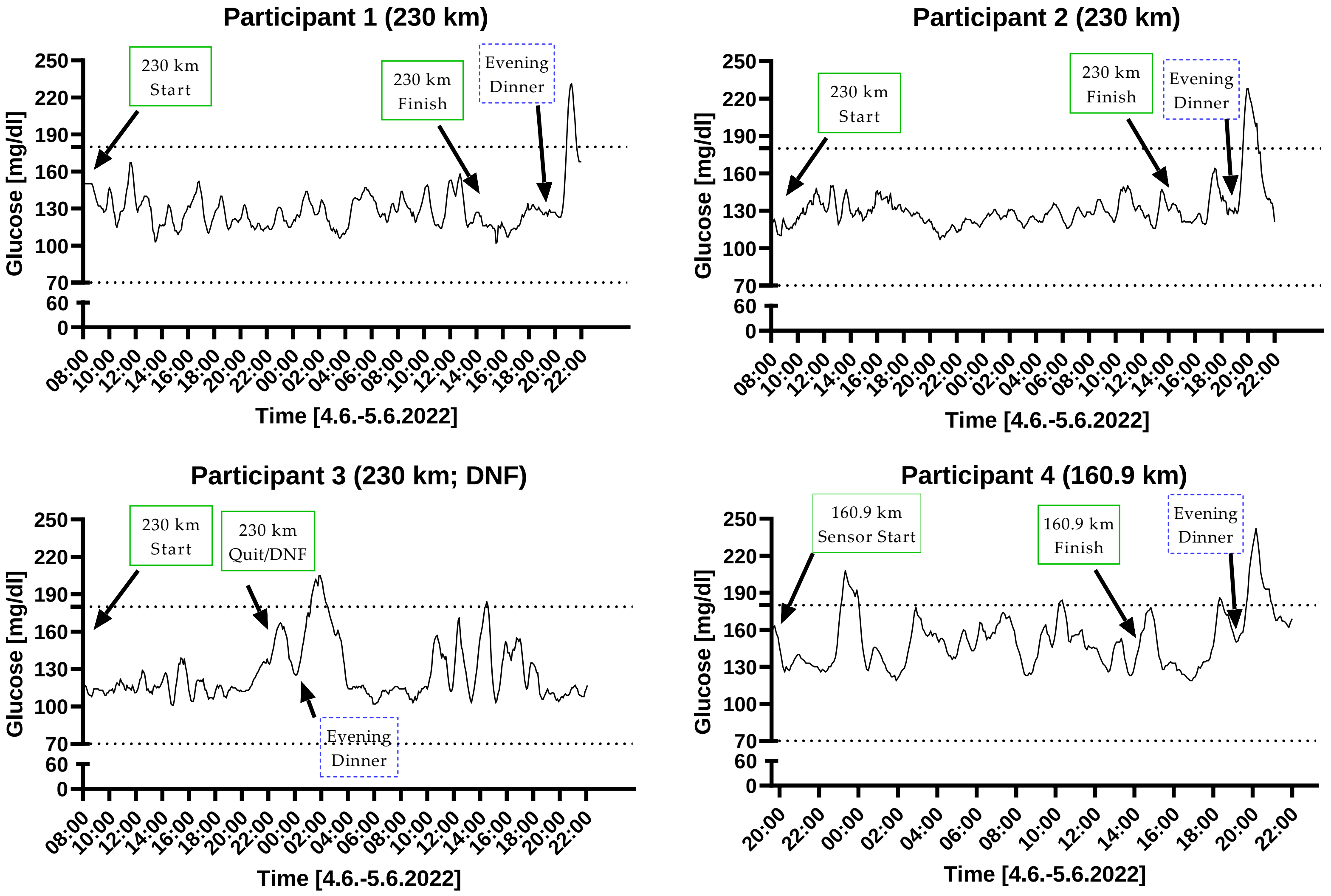

Figure S2: Intracutaneous glucose sensor measurements of four participants during the TorTour de Ruhr 2022. Dotted lines signalize the reference values set by the Free style libre 3 of 70 – 180 mg/dl. All participants 1-3 (two finishers, one did not finish DNF) running the 230 km distance stayed in the reference range during the race. Dinner after the race resulted in increases above the range. Participant 4 running the 160.9 showed higher values during the race compared to the 230 km runners but a similar fast glucose uptake into the cells.
