# Supplementary material for: Running for Your Life: Metabolic Effects of a 160.9/230 km Non-Stop Ultramarathon Race on Body Composition, Inflammation, Heart Function, and Nutritional Parameters
Source: Metabolites. 2022 Nov 18;12(11):1138. doi: 10.3390/metabo12111138 (PMC9692917; doi:10.3390/metabo12111138)
Supplement: Supplementary file 1 [file metabolites-12-01138-s001.zip › Supplemental S2.pdf]

**Supplemental S2: Echocardiographic data from a sub-sample of three participants before (Pre) and after (Post) finishing the 160.9 km /230 km ultramarathon TorTour de Ruhr 2022.**

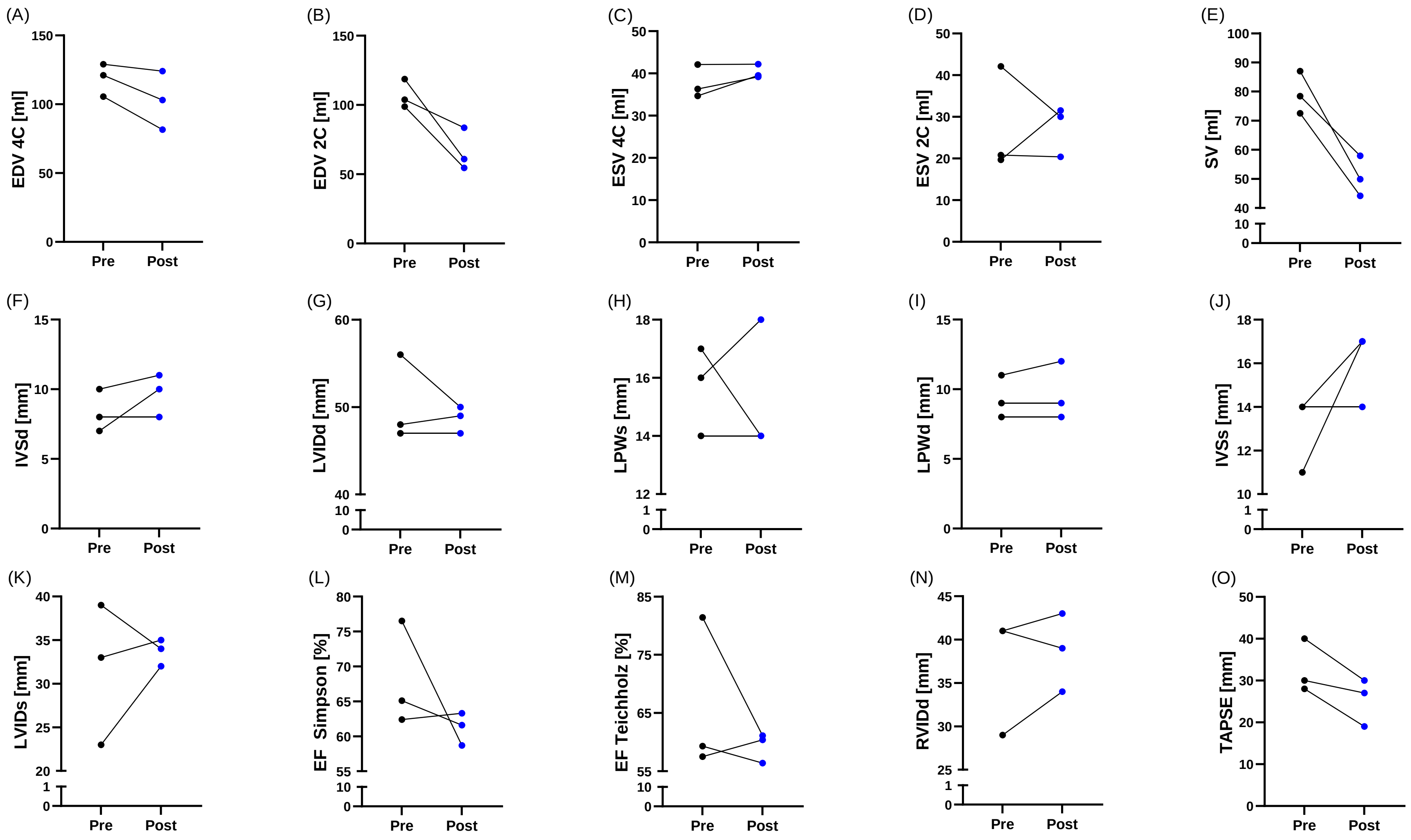


**Figure S1: The cardiac Pre to Post stress response obtained from three participants by echocardiography.** Individual changes are presented for (A) Enddiastolic Volume Four Chamber View (EDV 4C), (B) Enddiastolic Volume Two Chamber View (EDV 2C), (C) Endsystolic Volume Four Chamber View (ESV 4C), (D) Endsystolic Volume Two Chamber View (ESV 2C), (E) Stroke Volume (SV), (F) Interventricular Septum in end-diastole (IVSd), (G) Left ventricular internal diameter in end-diastole (LVIDd), (H) Left posterior wall end-systole (LPWs), (I) Left posterior wall in end-diastole (LPWd), (J) Interventricular Septum end-systole (IVSs), (K) Left ventricular internal diameter end-systole (LVIDs), (L) Ejection fraction by Simpson (EF Simpson), (M) Ejection fraction by Teichholz (EF T), (N) Right ventricular diameter in end-diastole (RVIDd) and (O) Tricuspid annular plane systolic excursion (TAPSE).

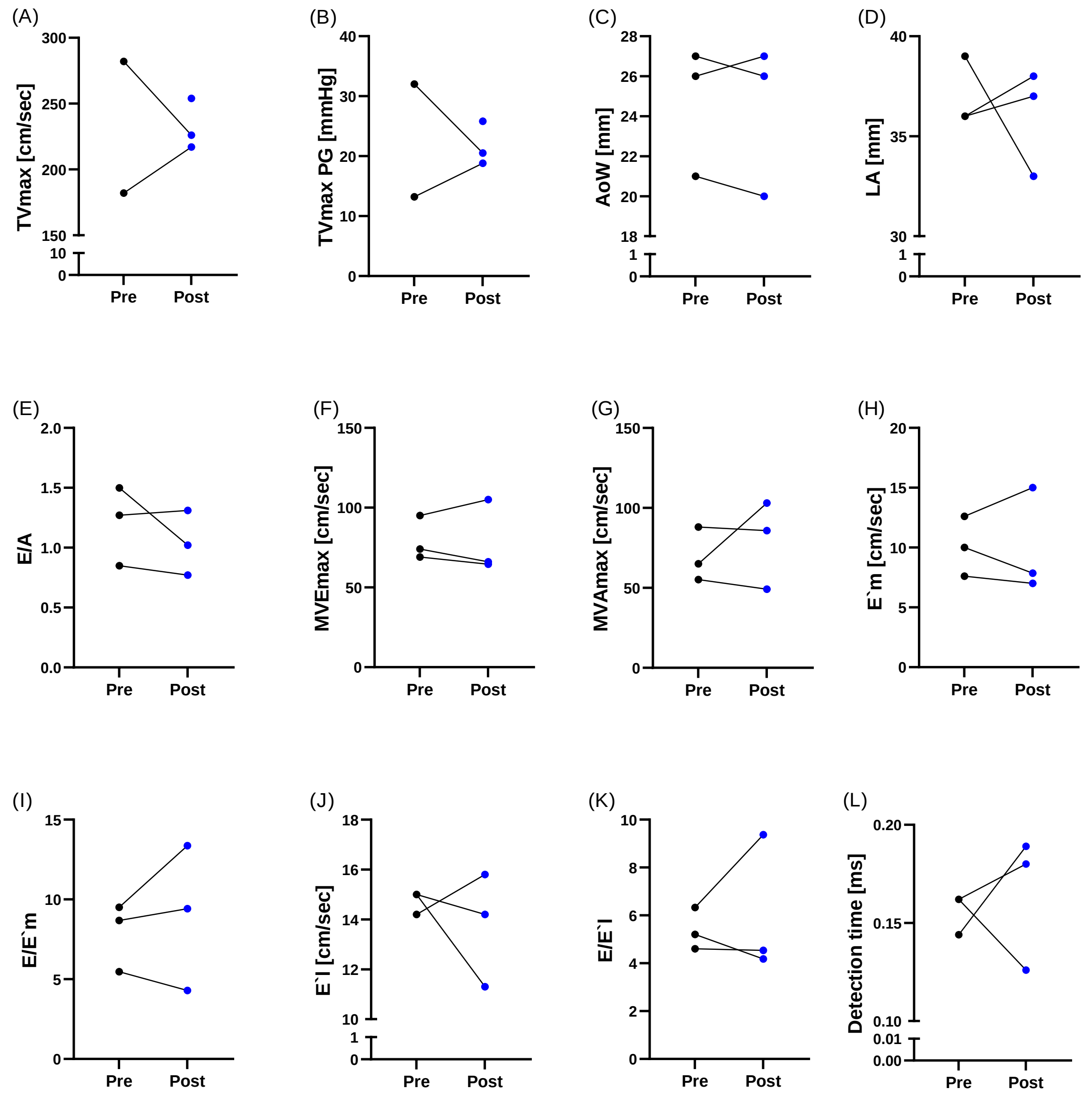


**Figure S2: The cardiac Pre to Post stress response obtained from three participants by echocardiography**. Individual changes are presented for (A) Tricuspid valve velocity (TVmax), (B) Tricuspid valve pressure gradient (TVmaxPG), (C) Aortic root diameter (AoW), (D) Left atrium diameter (LA), (E) E/A ratio, (F) Maximum velocities of E wave ($MVE_{max}$), (G) Maximum velocities of A wave ($MVA_{max}$), (H) Maximum myocardial velocities of the basal mitral annulus (E´m), (I) E/E`m ratio, (J) Maximum myocardial velocities of the lateral mitral annulus (E`l), (K) E/E`l ratio and (L) Deceleration Time (Dect Time).
